# Supplementary material for: Label-Free SERS Analysis of Serum Using Ag NPs/Cellulose Nanocrystal/Graphene Oxide Nanocomposite Film Substrate in Screening Colon Cancer
Source: Nanomaterials (Basel). 2023 Jan 13;13(2):334. doi: 10.3390/nano13020334 (PMC9864651; doi:10.3390/nano13020334)
Supplement: Supplementary file 1 [file nanomaterials-13-00334-s001.zip › nanomaterials-2144276-supplementary.pdf]

Supporting Information

Label-Free SERS Analysis of Serum Using Ag NPs/Cellulose Nanocrystal/Graphene Oxide Nanocomposite Film Substrate in Screening Colon Cancer

Jie Li [1,†], Qiutian She [1,†], Wenxi Wang [1], Ru Liu [1], Ruiyun You [1,*], Yaling Wu [2], Jingzheng Weng [1], Yunzhen Liu [1] and Yudong Lu [1,*]

[1] Fujian Provincial Key Laboratory of Advanced Oriented Chemical Engineer, Fujian Key Laboratory of Polymer Materials, College of Chemistry and Materials Science, Fujian Normal University, Fuzhou 350007, China

[2] College of Materials and Chemical Engineering, Institute of Oceanography Minjiang University, Fuzhou 350108, China

* Correspondence: youruiyun@fjnu.edu.cn (R.Y.); luyd@fjnu.edu.cn (Y.L.)

† These authors contributed equally to this work.

1. Introduction to the PCA-LDA model

PCA is known as Principal Component Analysis. PCA is a method of simplifying data structures by reducing dimensionality, specifically by transforming multiple variables into a small number of composite variables which in turn reflect most of the information of multiple variables [1,2].

Linear Discriminant Analysis (LDA) is a classical algorithm for pattern recognition. The basic idea of linear discriminative analysis is to project high-dimensional pattern samples into the best discriminative vector space, which has the effect of compressing the dimensionality of the feature space for extracting categorical information. The projection ensures that the pattern samples have the maximum inter-class distance and the minimum intra-class distance in the new subspace, meaning that the patterns have the best separability in that space [3–5].

PCA-LDA combination is primarily intended to address the transitional fitting of LDA during data processing. In order to solve this problem, a normalization of the problem is required. One approach is to first use PCA to reduce the dimensionality of the diagnostic discrimination and to retain the main features of the diagnostic information at the same time as clearly and intuitively defining the gaps between the various diagnoses [5,6].

2. ICP-OES analysis of silver in Ag NPs/CNC/GO nanocomposite film

Inductively coupled plasma optical emission spectroscopy (ICP-OES, Agilent 720ES, USA) was used to analyze the silver content of Ag NPs/CNC/GO nanocomposite film. Specifically, 0.5 cm × 1 cm Ag NPs/CNC/GO nanocomposite film was fully immersed in 20 mL of 2.0wt% HNO_3 solution and stood to completely dissolve all Ag NPs on the membrane surface overnight. A series of Ag NO_3 solutions (20.0 mg/L, 10.0 mg/L, 1.0 mg/L, 0.10 mg/L, 0.010 mg/L) in a volume of 10 mL were all configured to act as a reference baseline for measuring Ag NPs on the film. To better measure, all solutions were filtered using 0.22 μL filter membranes.

We have tested the Ag content of three synthesised Ag NPs/CNC/GO nanocomposite film in parallel using ICP-OES. The calculation equation is as follows:

$$\boldsymbol{C_X}(\mathbf{mg/cm^2}) = \frac{\boldsymbol{C_0}\,(\mathbf{mg/L}) \times \boldsymbol{f} \times \boldsymbol{V_0}(\mathbf{mL}) \times \mathbf{10^{-3}}}{\boldsymbol{m}(\mathbf{cm^2}) \times \mathbf{10^{-3}}}$$

Where $\boldsymbol{C_0}$ represented the concentration of the elements in solution after the Ag NPs/CNC/GO nanocomposite film was dissolved by nitric acid; $\boldsymbol{V_0}$ was the volume of the fixed volume of the composite membrane after dissolution; $\boldsymbol{f}$: dilution multiple; $\boldsymbol{m}$

represented the area of the nanocomposite membrane taken for the analysis of the sample. C_X was the concentration of the element Ag contained in the membrane.

Table S1. ICP-OES data on the concentration of silver contained in Ag NPs/CNC/GO nanocomposite film.

Number	m (cm²)	V_0 (mL)	Test elements	C_0 (mg/L)	f	C_X (mg/cm²)	C_X (μg/cm²)
1	0.5	10	Ag	4.91	1	0.10	98.27
2	0.5	10	Ag	4.92	1	0.10	98.33
3	0.5	10	Ag	4.91	1	0.10	98.30

3. Hydrophobic characterization of Ag NPs/CNC/GO nanocomposite film

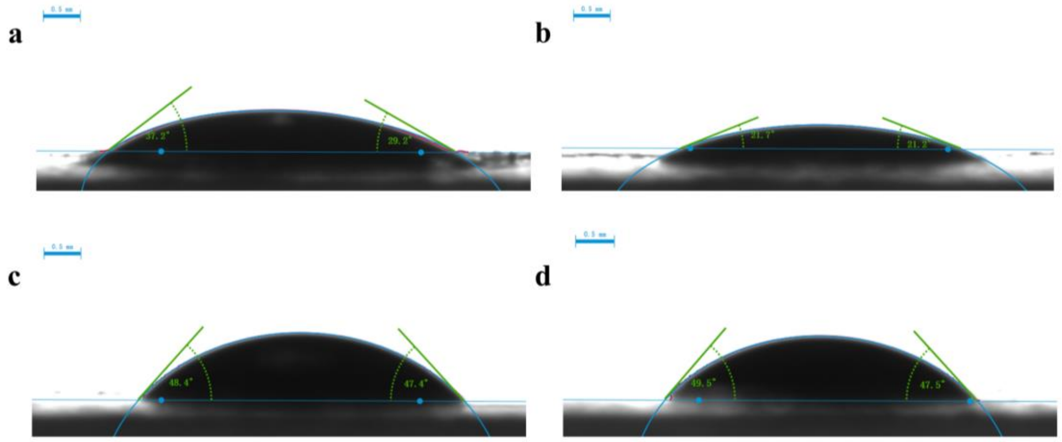

Figure S1. Water contact angle of the membranes. (a): CNC-BCM; (b): GO-BCM; (c): Ag NPs/CNC-BCM; (d): Ag NPs/CNC/GO nanocomposite film.

We have obtained the hydrophobic properties of the four films by a series of measurements of the water contact angle of 2 μL droplets in the environment (DSA25, Kurss, Germany). In Fig. S1(a) and S1(b), we can clearly see that the water contact angle of CNC is around 30°, and GO presents a much lower contact angle, both of which exhibit super hydrophilicity [7]. It is due to the fact that cellulose molecules contain a large number of polar groups and have a great affinity for water. Loaded silver composite films showed an opposite phenomenon of increasing water contact angle, as shown in Fig. S1(c) and S1(d). Due to the hydrophilic nature of Ag NPs, the hydrophilicity of CNC and GO deposited with Ag NPs ought to increase, however, the opposite results were obtained at this point, mainly owing to the fact that the density of Ag NPs was increased by using centrifugation, resuspension, and centrifugation when spin-coating our suspensions on BCM, and so our prepared composite films showed a decrease in hydrophilicity. Finally, Ag NPs/CNC/GO nanocomposite film exhibits strong hydrophilic properties.

4. Conditions Optimization

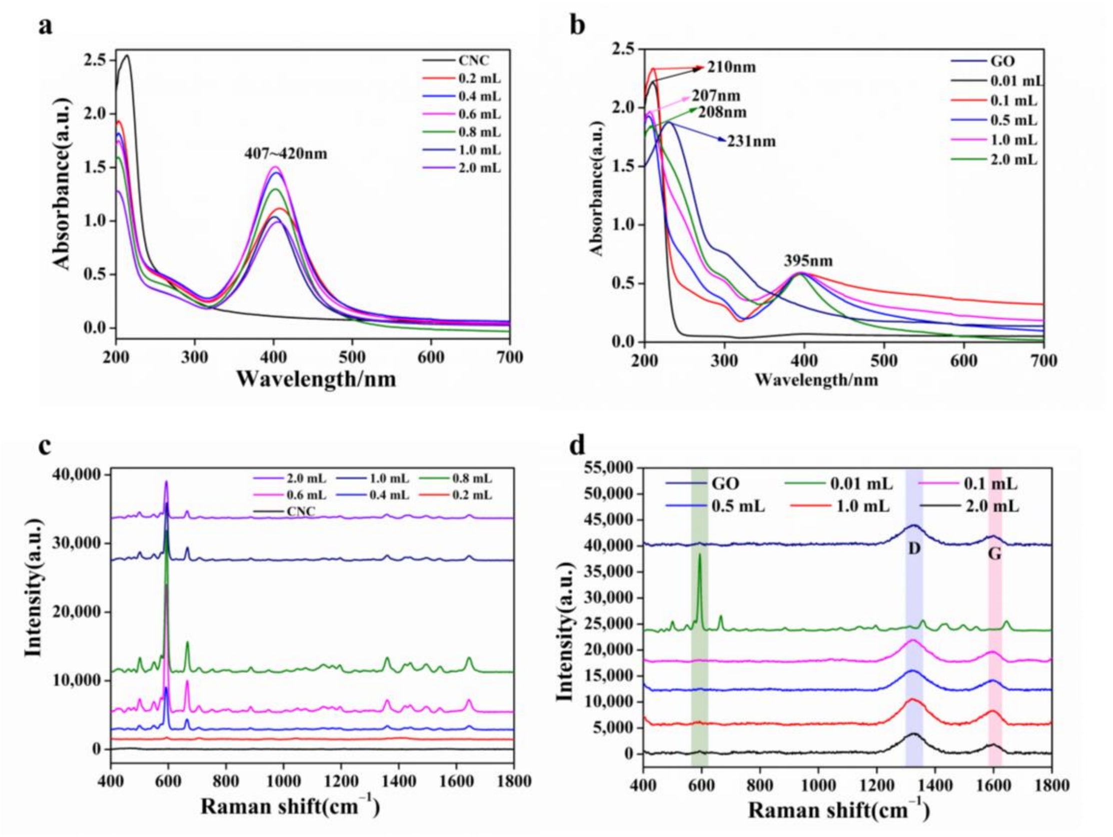

Figure S2. UV-Vis absorption spectra of (a): Ag NPs/CNC substrates synthesized by adjusting the volume of $NaBH_4$ (0 mL, 0.2 mL, 0.4 mL, 0.6 mL, 0.8 mL, 1.0 mL and 2.0 mL); (b): the content of GO dispersions (0.01 mL, 0.1 mL, 0.5 mL, 1 mL, 2 mL) added to the Ag NPs/CNC substrates. (c) and (d): SERS spectra of the substrates corresponding to (a) and (b), respectively.

5. Structure of bacterial cellulose membrane (BCM)

a

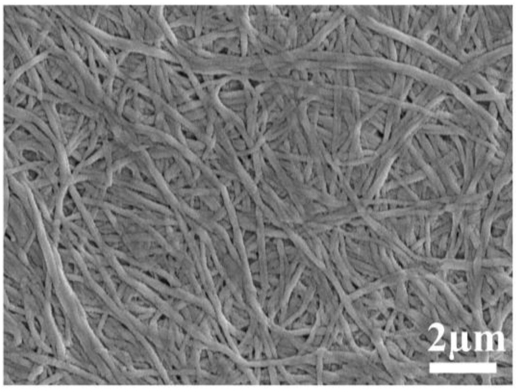

Figure S3. (a): SEM image of BCM.

6. Ag NPs/CNC-BCM sensitivity

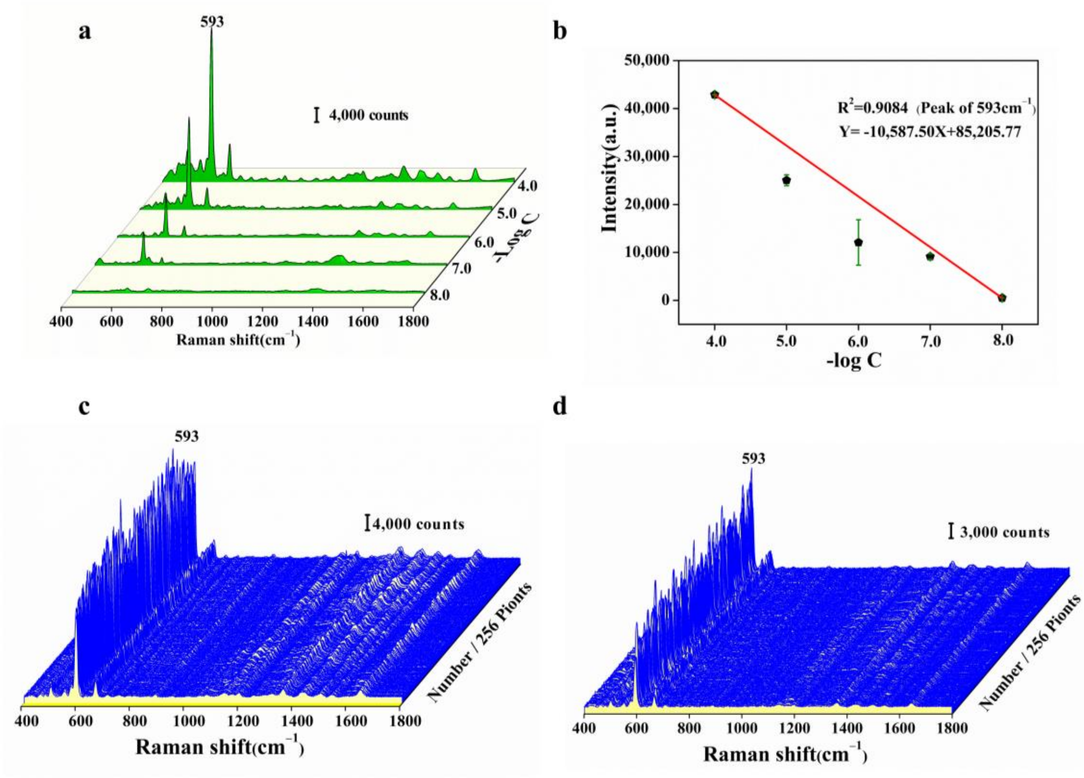

Figure S4. (a): the SERS spectra of CNC-Ag NPs-BCM obtained by adsorption of different concentrations (10^{-4} M-10^{-8} M) of NBA onto 0.8 mL $NaBH_4$; (b): A linear curve of the SERS intensity versus concentration at 593 cm^{-1} corresponding to (a). (c) and (d) were waterfall plots of the SERS spectra of Ag NPs/CNC/GO nanocomposite film and Ag NPs/CNC-BCM adsorbed with 10^{-5} M NBA in the field of 30 μm × 30 μm array respectively.

7. Ag NPs/CNC/GO nanocomposite film to measure the uniformity of serum samples

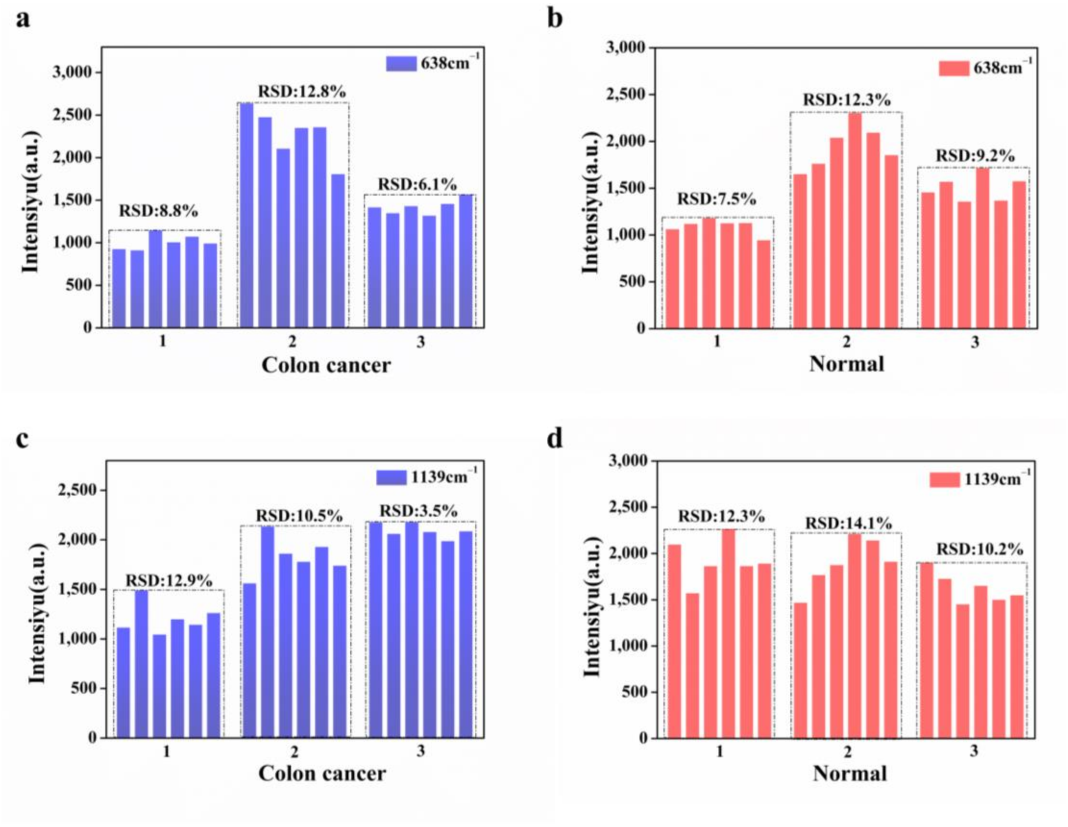

Figure S5. (a), (c): Histograms of the SERS intensity of three randomly selected cases in the colon cancer groups with their corresponding characteristic peaks at 638 and 1139cm^{-1}, where each case was tested six times in parallel; (b), (d): Histograms of the SERS intensity of three randomly selected cases in the normal groups with their corresponding characteristic peaks at 638 and 1139cm^{-1}, where each case was tested six times in parallel.

8. Spectrum peak attribution

Table S2. SERS spectral peak positions and tentative peak attribution.

Peak position (cm^{-1})	Tentative biomolecular assignments [8–10]
494	Polysaccharides (amylase, branched chain starch)
595	Amide VI, Ascorbic acid
636	L-tyrosine
726	DNA
810	Glutathione, L-serine
889	D-galactosamine, Glutathione
957	Alpha-helix proline, Valine
1014	Phenylalanine
1068	Collagen
1101	Phenylalanine
1135	Deoxyribose phosphate
1208	L-tryptophan
1334	Nucleic acid bases
1582	Pyrimidine ring (nucleic acid) and hemoglobin
1685	Amino compound I

References

1. N. B.; Linford, M. R., A perspective on two chemometrics tools: PCA and MCR, and introduction of a new one: Pattern recognition entropy (PRE), as applied to XPS and ToF-SIMS depth profiles of organic and inorganic materials. *Applied Surface Science* **2018,** *433*, 994-1017.
2. Zare, A.; Ozdemir, A.; Iwen, M. A.; Aviyente, S., Extension of PCA to Higher Order Data Structures: An Introduction to Tensors, Tensor Decompositions, and Tensor PCA. *arXiv e-prints* **2018**.
3. Li, X.; Zhang, Y.; Zhang, R., Self-Weighted Unsupervised LDA. *IEEE Transactions on Neural Networks and Learning Systems* **2021**, 1-6.
4. Zhu, F.; Gao, J.; Yang, J.; Ye, N., Neighborhood linear discriminant analysis. *Pattern Recognition* **2022,** *123*, 108422.
5. Kim, D. H.; Song, B. C., Virtual sample-based deep metric learning using discriminant analysis. *Pattern Recognition* **2021,** *110*, 107643.
6. Lasalvia, M.; Capozzi, V.; Perna, G. A Comparison of PCA-LDA and PLS-DA Techniques for Classification of Vibrational Spectra *Applied Sciences* [Online], 2022.
7. Chen, G.; Chen, T.; Hou, K.; Ma, W.; Tebyetekerwa, M.; Cheng, Y.; Weng, W.; Zhu, M., Robust, hydrophilic graphene/cellulose nanocrystal fiber-based electrode with high capacitive performance and conductivity. *Carbon* **2018,** *127*, 218-227.
8. Li, X.; Yang, T.; Li, S.; Wang, D.; Guan, D., Detecting Esophageal Cancer Using Surface-Enhanced Raman Spectroscopy (SERS) of Serum Coupled with Hierarchical Cluster Analysis and Principal Component Analysis. *Applied Spectroscopy* **2015,** *69* (11), 1334-1341.
9. Li, H.; Zhang, S.; Zhu, R.; Zhou, Z.; Xia, L.; Lin, H.; Chen, S., Early assessment of chemotherapeutic response in hepatocellular carcinoma based on serum surface-enhanced Raman spectroscopy. *Spectrochimica Acta Part A: Molecular and Biomolecular Spectroscopy* **2022,** *278*, 121314.
10. Lin, C.; Liang, S.; Li, Y.; Peng, Y.; Huang, Z.; Li, Z.; Yang, Y.; Luo, X., Localized plasmonic sensor for direct identifying lung and colon cancer from the blood. *Biosensors and Bioelectronics* **2022,** *211*, 114372.
